# DJ1 at the interface between neuro-degeneration and cancer

**DOI:** 10.18632/oncotarget.14889

**Published:** 2017-01-29

**Authors:** Johannes Meiser, Alexei Vazquez, Karsten Hiller

**Affiliations:** Braunschweig Integrated Centre of Systems Biology, University of Braunschweig, Braunschweig, Germany

**Keywords:** neuro-degeneration, cancer, DJ1, cellular metabolism, redox homeostatsis, Neuroscience

DJ-1 (*PARK7*) was first identified as an oncogene that represses PTEN and thus induces proliferation in certain types of cancer [1]. Later, it was found that loss of DJ-1 leads to early onset of Parkinson’s disease, characterised by a loss of dopaminergic neurons in the midbrain [2]. Even after more than a decade of intense research, the exact cellular function of DJ-1 is still elusive. Two hallmarks of DJ-1 are scavenging of reactive oxygen species (ROS) and its relation to mitochondria, a hub for central metabolism. Even though highly proliferating cancer cells and post-mitotic neurons are very different cell types, they have a common challenge: balancing ROS, derived from high metabolic burden.

In a recent study by our lab, we investigated the role of DJ-1 on cellular metabolism in post-mitotic human neurons derived from the midbrain [3]. In line with the common observation of increased sensitivity to ROS, we observed an increased GSSG (oxidised glutathione) to GSH (reduced glutathione) ratio and increased sensitivity to the ROS inducing compound 6-hydroxydopamine, an oxidation product of dopamine. Our results suggest that altered glutamine and serine metabolism might be the metabolic cause for the observed changes in GSH homeostasis. Glutathione consists of the three amino acids glutamate, glycine and cysteine. Glutamate is derived from glutamine and glycine and cysteine can be derived from serine by the activity of serine hydroxymethyltransferase (SHMT) and via the transsulfuration pathway, respectively. The SHMT reaction also fuels the folate mediated one-carbon (1C) metabolism by donating carbon three of serine to tetrahydrofolate (THF). 5,10-methylene-THF can then be oxidised to formate via the folate cycle. Depending on the enzyme isoform and the compartment, this oxidation is NADP+ or NAD+ dependent, providing additional reducing potential to the cell [4]. While NADPH can be used to reduce GSSG back to GSH, NADH can be oxidised via oxidative phosphorylation to generate ATP.

Our work provides a comprehensive overview of the effects of loss of DJ1 on central metabolism in non-proliferating neuronal cells and points to key metabolic steps that are crucial for cellular ROS homeostasis. However, the mechanistic link, how DJ-1 controls glutamine and serine-1C metabolism is still under investigation. In cancer cells, the serine synthesis pathway (SSP) and cellular antioxidant responses are, amongst others, controlled by NRF2 that can be stabilised by DJ-1 [5]. However, we observed that NRF2 is in very low abundance in our neuronal cell model. This is interesting for two reasons: 1) This finding emphasises the importance of studying mechanistic links individually in different cell models and that one has to be very careful in drawing general conclusions based on data derived from one single cell model. 2) The low abundance of NRF2 in the neuronal cell model raises the question of how SSP, glutamine and 1C metabolism is regulated by DJ-1. Based on a previous study, together with our collaborators from the Leist lab we have strong evidence that such a candidate could be ATF4 [6]. In the latter study we investigated the cellular events in neuronal cells upon increased oxidative stress. Upon elevated, but non-apoptotic oxidative stress the SSP, transsulfuration pathway and 1C metabolism was up regulated and centrally controlled by ATF4. Concluding from our finding that loss of DJ-1 affects serine and 1C metabolism, that loss of DJ-1 makes cells more susceptible to oxidative stress [3] and that neuronal cells exposed to oxidative stress induce ATF4 to increase serine biosynthesis, transsulfuration and 1C metabolism [6], is suggestive that DJ-1 might control these metabolic pathways via ATF4 (Figure 1). However, this is still under investigation.

As we mentioned in the introduction, DJ-1 is associated with mitochondria. A canonical function of mitochondria, especially in non-proliferating cells, is the provision of ATP to fulfil the energy demands of these highly catabolic cells. Intriguingly, we demonstrated very recently that the function of serine mediated 1C metabolism is not only to fulfil anabolic demands but that the major fraction of serine has a catabolic role. In contrast with current understanding of cancer metabolism [7], we have demonstrated that most of the serine, which is the major carbon donor of 1C metabolism, is catabolised to glycine and formate in the mitochondria and then released by cells [8]. Moreover, we have demonstrated that the oxidation of 5,10-methylene THF is coupled to complex I in the mitochondria and thus coupled to ATP generation. In addition, the release of every formate contributes also with net ATP production to the cellular energy pool. These observations have been made in proliferating cancer and non-transformed, proliferating fibroblast cell lines. Based on our findings that DJ-1 affects serine 1C metabolism in non-proliferating, highly catabolic neuronal cells raises the question of whether DJ-1 also affects formate release in neurons and if serine catabolism via the folate pathway might be an alternative way for energy supply in neurons. This notion could also explain our observed differences in mitochondrial motility upon DJ-1 silencing.

In summary, this work underlines the exciting but at the same time challenging topic surrounding the still elusive functions of DJ-1, a protein that apparently has to be at the right dose in our cells to maintain cellular integrity.

## References

[1] Kim RH (2005). Cancer Cell.

[2] Bonifati V (2003). Neurol Sci.

[3] Meiser J (2016). Neurobiol Dis.

[4] Tibbetts AS (2010). Annu Rev Nutr.

[5] Clements CM (2006). Proc Natl Acad Sci U S A.

[6] Krug AK (2014). Cell Death Dis.

[7] Meiser J (2016). FEBS J.

[8] Meiser J (2016). Sci Adv.

